# The CIRCLE Care Home Guide: A Co‐Designed Resource on LGBTQ+ Inclusion for Care Homes

**DOI:** 10.1111/hex.70309

**Published:** 2025-08-13

**Authors:** Jolie R. Keemink, John Hammond, Grace Collins, Joseph Price, Martin Wells, Sallie Johnson, Susan A. Rugg, Martin Parish, Andrew King, Kathryn Almack

**Affiliations:** ^1^ University of Kent Canterbury UK; ^2^ PPI Member Canterbury UK; ^3^ University of Surrey Guildford UK; ^4^ University of Hertfordshire Hatfield UK

**Keywords:** care homes, co‐design, LGBTQ+ inclusion, resource

## Abstract

**Background:**

Many older LGBTQ+ people still face discrimination in care home settings, and many care home staff do not have the knowledge required to provide LGBTQ+ inclusive care. Despite the availability of valuable resources on LGBTQ+ inclusion, its widespread use in social care practice in the United Kingdom is lacking. To address this issue, the current study presents a novel resource on LGBTQ+ inclusion co‐designed by older LGBTQ+ people, care home staff and researchers, developed to be usable in a care home context.

**Methods:**

Five older LGBTQ+ people, four care home staff and three researchers co‐designed the novel resource in four online co‐design meetings using an adapted experience‐based co‐design process. Co‐design meetings were recorded to collect data on the co‐design process. We organised an online focus group to collect data on how the co‐design members had experienced the co‐design process and their thoughts on the anticipated impact of the novel resource.

**Results:**

The co‐design group successfully developed a resource that adds to existing resources by being fully co‐designed by older LGBTQ+ people, care home staff and researchers. It prioritises positive actions care staff can take along someone's care journey, available in formats designed to work well in a care home environment. Further, the focus group data highlighted the power of lived experience, from perspectives of both older LGBTQ+ people and care staff. Both perspectives brought unique elements to the co‐design sessions and are represented in the final resource.

**Conclusion:**

Through an inclusive co‐design process, we developed a new resource for care homes to support LGBTQ+ inclusive practice. Our findings demonstrate the importance of collaboration between older LGBTQ+ people and care home staff to develop resources for inclusive practice. The final version of the resource has been launched and future research will assess its impact in practice.

**Patient and Public Involvement:**

The resource was fully co‐designed by older LGBTQ+ people and care home staff. The wider project was supported by an older LGBTQ+ person as co‐applicant.

## 1 Introduction

The aim of this paper is threefold: (1) to introduce a novel, co‐designed resource on inclusive care for older lesbian, gay, bisexual, trans and queer (LGBTQ+) people for use in care homes, (2) to describe the process of co‐designing this resource and (3) to present data on the perspectives of co‐design members on the co‐design process and outcome.

Many staff working in residential care in the United Kingdom lack the awareness, knowledge and skills to provide inclusive care to older LGBTQ+ people [1, 2, 3, 4]. Despite reportedly having largely positive attitudes towards LGBTQ+ residents, many staff do not recognise the relevance of LGBTQ+ status for someone's care needs, which is often expressed in statements such as: ‘Sexuality or gender does not matter. We treat everyone the same’ ([2], p. 187). Whilst this sentiment is usually well‐meant, it presumes a heteronormative and cis‐normative approach to care and disregards the unique experiences and challenges of older LGBTQ+ people, rendering this population invisible [5, 6, 7, 8].

Many LGBTQ+ people report experiences of discrimination and barriers in access to care services [3, 9]. When considering residential care, many describe fears of having to hide their identity and ‘go back into the closet’ to be safe [10, 11, 12, 13]. There is also evidence of concerningly high rates of reported LGBTQ+ discrimination in care homes, often not followed up by authorities [14]. The barriers in access to inclusive residential care are characteristic of the lifelong discrimination the older LGBTQ+ population has faced, which has severely affected their confidence in public services and their comfort to disclose their LGBTQ+ identity [10, 12]. At the same time, the accumulation of discriminatory experiences has led to an increased likelihood of health and care needs because of minority stress [15, 16]. Minority stress theory proposes that members of a minority group experience unique stressors contributing to poor physical and mental health [17]. Furthermore, older LGBTQ+ people may rely more heavily on social care services because they are less likely to have supportive relationships with their family of origin [18, 19]. This juxtaposition of higher care needs and greater access barriers, neither of which are adequately recognised, underlines the urgent need for improvements with regard to LGBTQ+ inclusion within residential care. The current study aimed to co‐design a practical and usable resource on *how to* make care homes more LGBTQ+ inclusive that is implementable across the sector.

Whilst the UK government has recognised a gap in service provision [20, 21], there is currently no unified approach or national guidance to support care home settings in improving their provision of LGBTQ+ inclusive care. Hunt et al. [22] reviewed educational materials on LGBTQ+ inclusion for UK health and social care staff that have been available since 2010. More recently, further resources were published, including Care Under the Rainbow [23], Confident with Difference [24], safe to be me [25] and the LGBTQ+ Learning Framework [26]. Despite the availability of these resources, older LGBTQ+ people continue to report experiences of discrimination, and the use of educational materials in practice seems limited and anecdotal. Hunt et al. [22] attribute the lack of use in practice to the fact that knowledge transfer in itself is insufficient to change practice, citing Wensing et al. [27], who report that knowledge transfer is necessary but not sufficient. Whilst they are informative, valuable and necessary, previous resources fail to adequately include techniques that support the intended audience to change their behaviour. The actionable, practical *how* of LGBTQ+ inclusion in care settings is not always clearly included. Moreover, only a handful of resources were specifically designed for care home settings (e.g. Care Under the Rainbow from [23]), and whilst these involved older LGBTQ+ people and care staff, none of these report direct and continuous collaboration between care staff and LGBTQ+ people in resource development, which is arguably essential for relevance and usability. Additionally, previous resources have been predominantly presented in PDF or video format and do not explicitly report considering formats preferred by the target audience.

From an implementation perspective, it is important to recognise the barriers to using educational resources in practice. Previous research highlights that inadequate staffing, austerity and lack of applicability are important barriers to implementing guidelines in care homes [28]. Some of the available resources are lengthy and take ample time and effort to engage with. For example, the elaborate LGBTQ+ Learning Framework [26] is 90 pages long. In a context of austerity and unparalleled workforce pressures [29], it is unlikely that frontline care home staff will engage with time‐intensive educational materials, especially when it comes to the neglected topic of sexuality and gender [30, 31]. The resource presented in this paper attempted to improve implementation success and support behaviour change by prioritising actions rather than knowledge transfer, by highlighting lived experience (from both care staff and older LGBTQ+ people) through co‐design and by using formats preferred by the target audience.

LGBTQ+ older people have been largely overlooked in consultations on and the development of LGBTQ+ inclusive care services and educational materials [32, 33, 34, 35], and very few examples have been reported in the academic literature. Co‐creation of resources and services with care homes is more widespread in the United Kingdom [36], but only one example involves collaboration between care home staff and older LGBTQ+ people co‐producing research [37]. Several studies have highlighted the importance of facilitating communication and collaboration between older LGBTQ+ people and care organisations for the creation of affirmative and culturally responsive education materials [11, 38, 39]. Educational material on LGTBQ+ inclusion is more likely to be effective and usable if it is developed by all stakeholders concerned. Older LGBTQ+ people are vital in this process to address the key elements of inclusion and *how* these are achieved. Care home staff are essential to provide insights on the type of resource and content would be most engaging for the workforce. The current study is the first to report on the co‐design of a novel, free resource on LGBTQ+ inclusion for care homes. This study was part of a wider project called CIRCLE (Creating Inclusive Residential Care for LGBTQ+ Elders), examining multiple ways of supporting care homes to improve their LGBTQ+ inclusive care practice.

## 2 Materials and Methods

### 2.1 Participants and Recruitment

We aimed to recruit five older LGBTQ+ people and five care home staff for our co‐design group. Group size was based on previous literature [40, 41]. For all participants, the inclusion criteria comprised: interested in making care homes more inclusive and available for five online meetings between October 2023 and March 2024. For the older LGBTQ+ group specifically, inclusion criteria were: aged 50+ and identifying as LGBTQ+. The age category was decided on with the study advisory group. Interested care home staff could be employed in any type of role within a care home, and there were no criteria around their sexuality, gender or age.

Recruitment of older LGBTQ+ people occurred through existing links with LGBTQ+ voluntary organisations, which shared a recruitment advert with members. Recruitment of care home staff was pursued through local authorities, contacts sharing the advert in local care homes and newsletters. The recruitment advert was also shared on social media channels and through the project advisory group. Interested participants got in touch via email. They were then offered a brief online conversation to discuss the project and the details of involvement. Eleven people came forward, but two (one care home staff and one older LGBTQ+ person) withdrew because of conflicting time commitments. The remaining nine people were included in the co‐design group.

Four older LGBTQ+ people joined the co‐design meetings. One additional older LGBTQ+ person preferred to provide input on a one‐to‐one basis. Four care home staff attended the co‐design meetings. Three were managers, and one was care quality director. Three researchers attended the co‐design meetings, and a fourth researcher was present during a later online focus group evaluating the co‐design process. Tables 1, 2, 3 provide an overview of the demographic characteristics of the people involved in the co‐design work, including the research team.

**Table 1:** Demographic characteristics of older LGBTQ+ co‐design members.

Demographic characteristic	*N* (total = 5)
Sexual orientation	
Lesbian	3
Gay	1
Queer	1
Gender identity	
Cis woman	2
Cis man	2
Non‐binary	1
Age	
61–65	1
66–70	4
Ethnicity	
White British/White Other	5
Religion	
Christian	1
No religion	4
Disability	
Yes	2
No	3
Care experience	
Cares/cared for a partner or family member	4
Has received social care	2

**Table 2:** Demographic characteristics of care home staff co‐design members.

Demographic characteristic	*N* (total = 4)
Sexual orientation	
Heterosexual	4
Gender identity	
Cis woman	4
Age	
46–50	3
61–65	1
Ethnicity	
White British/White Other	3
Black/African/Caribbean/Black British	1
Religion	
No religion	4
Disability	
Yes	0
No	4
Professional experience in adult social care	
6–10 years	1
More than 10 years	3

**Table 3:** Demographic characteristics of researcher co‐design members.

Demographic characteristic	*N* (total = 4)
Sexual orientation	
Gay	2
Queer	1
Heterosexual	1
Gender identity	
Cis woman	2
Cis man	2
Age	
21–25	1
31–35	2
51–55	1
Ethnicity	
White British/White Other	4
Religion	
No religion	4
Disability	
Yes	1
No	3
Care experience	
Cares/cared for a partner or family member	1
Has received social care	3

Older LGBTQ+ people received £25 per hour (£50 per co‐design meeting), in accordance with NIHR INVOLVE payment standards [42]. Care providers joined the meetings in work time and received a one‐off £50 voucher as a thank you for their involvement.

### 2.2 Design

The research team adopted a co‐design approach for resource development. We followed the definition of co‐design described by Robert et al. [43]: Co‐design is a distinct creative process, where experts by experience and care staff work in partnership to improve services or develop interventions. We specifically used the experience‐based co‐design (EBCD) method [44], a participatory approach combining participant‐centred orientation (emphasising lived experience) with a collaborative change process (co‐design). The EBCD method is well‐suited to bring together two groups that potentially have (historical) tension between them and has been used successfully to co‐design resources for practice [45, 46]. We used an adapted EBCD process and applied relevant elements within the time frame and resources available.

**Figure 1:**
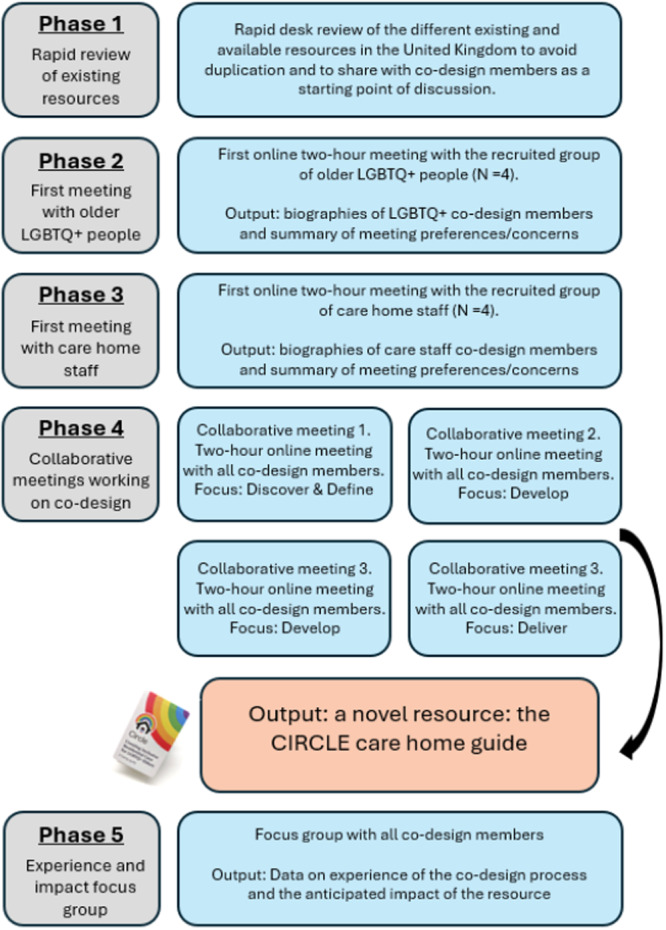
Overview of the co‐design process.

A key element of EBCD is to first organise separate meetings for experts by experience and for care staff and subsequently bring them together in collaborative meetings to work on co‐designing a solution for an identified issue. The purpose of the separate meetings is to create a safe environment where individual groups can share their experiences and potential concerns leading up to the co‐design process. The EBCD process was carried out online on Zoom [47] to aid accessibility in terms of employment commitments, geographical location and physical ability. The research team took turns to facilitate sessions. We viewed ourselves as co‐design members equal to recruited members. We were not prescriptive about the content and form of the resource; we only provided pragmatic boundaries on finances and time. The process in the current study followed five phases, described in more detail below. Figure 1 presents a visual overview of the phases followed in this study.

#### 2.2.1 Phase 1: Rapid Review of Existing Resources (September 2023)

In the literature review for the funding application for this project, the research team, with input from a co‐applicant with lived experience, had already identified the need for a more action‐oriented, care‐home‐specific, co‐designed resource. We carried out an additional rapid desk search in September/October 2023 to explore whether further resources had been published since. The rapid review focused on UK‐produced resources on LGBTQ+ inclusion for older people in social care. We did not use a formal method, but did specify a time frame (2022–2023), and used variations on ‘LGBTQ+ inclusion in care’ in Google and Google Scholar. The key findings of this desk review are discussed in Section 1. A summary of the resources was produced to present to co‐design members in the first meetings to inform their judgements about what the new resource could add to existing materials.

#### 2.2.2 Phase 2: Meeting With Older LGBTQ+ People (October 2023)

The first online co‐design meeting involved four older LGBTQ+ people and three members of the research team. The main aims of this meeting were: (1) to establish rapport, (2) to discuss expectations, (3) to understand potential concerns around safety and inclusivity, (4) to discuss ideas for reaching consensus and (5) to introduce an overview of existing resources. The research team focused on creating an informal atmosphere where everyone felt comfortable to contribute.

#### 2.2.3 Phase 3: Meeting With Care Home Staff (November 2023)

This meeting involved four care home staff and three members of the research team. The main aims of the meeting corresponded with those described above.

#### 2.2.4 Phase 4: Collaborative Meetings (December 2023 to March 2024)

This phase consisted of four collaborative meetings in which co‐design members focused on co‐designing a new resource on LGBTQ+ inclusion for care homes. A digital artist was also present, who used the discussion to design the resource. We initially worked on creating group cohesion and emphasising our shared goal. We were guided by the co‐design stages as described by the Design Council [48] and reported in Robert et al. [43]: discover, define, develop and deliver. In the first collaborative meeting, we focused on discover and define, in which the issue (the lack of LGBTQ+ inclusion in residential care) was explored widely through divergent thinking. The discussions focused on understanding the problem and defining what type of new solution was needed. The following three meetings involved convergent thinking and focused on taking focused action.

#### 2.2.5 Phase 5: Focus Group (May 2024)

When the co‐design process had been completed, we organised a 1‐h online focus group with co‐design members to collect data on the perspectives of co‐design members on the co‐design process and outcome.

### 2.3 Data and Analysis

The data collected in this study comprised recordings and minutes of the collaborative co‐design sessions, as well as the recorded and transcribed content of the focus group.

#### 2.3.1 Co‐Design Sessions

Each co‐design session was recorded and minutes were shared with the co‐design group in advance of the next session and to use as data to report on the co‐design process. The research team watched the recordings of each collaborative session, took notes and came together to discuss the stages of resource development. These data have been used to summarise the process of resource development in Section 3.1.

#### 2.3.2 Focus Group

All co‐design group members were invited to attend an online focus group when the co‐design process had been completed. The three members who were not available for the focus group (two care home staff and one lived experiencer) answered the focus group questions in written form, which has been included in the analysis. The change in group size did not seem to alter the group dynamics. The discussion guide was focused on three matters: (1) the experience of co‐design members, (2) views on the final resource and (3) the anticipated impact of the final resource. Example questions include: How was it to be involved in the co‐design process? What are your thoughts on the final resource? What do you think the impact of the resource will be?

A member of the research team transcribed the focus group discussion verbatim. Transcripts were imported into NVivo for conventional content analysis [49], using inductive, iterative coding. Our analysis could be described as predominantly manifest, with occasional elements of inference, as we believed this is necessary to articulate the meaning of the data. The researchers familiarised themselves with the content by attentively reading through the data multiple times and taking notes on potential codes informed by the data. A coding table was used to iteratively assign (parts of) responses to the relevant content category, which informed the synthesis and interpretation of the data. A second researcher scrutinised the coding work for rigour. Disagreements were discussed until a consensus was reached.

### 2.4 Project Advisory Group

The wider project was supported by a project advisory group, including older LGBTQ+ people (*N* = 3), adult social care sector staff (*N* = 5), voluntary sector representatives (*N* = 3) and researchers (*N* = 3), who advised on all stages of the co‐design work and supported with recruitment.

### 2.5 Ethics

This study was approved by the Ethics Committee of the School for Social Policy, Sociology, and Social Research at the University of Kent in November 2022 (Ref: SCREA ID 0722). All participants provided written consent to be involved in the co‐design process, for the sessions to be recorded and for participation in the focus group. At the start of each session, all members were asked if they were still happy to be involved.

## 3 Results

In this section, we will first describe the stages of resource development and present the final resource (Aims 1 and 2 of the paper). Subsequently, we will present a content analysis of the focus group discussion (Aim 3 of the paper).

### 3.1 Co‐Design Meetings

#### 3.1.1 Bringing the Groups Together

In the initial, separate meetings, both groups mentioned the importance of kindness, respect, compassion, honesty and curiosity. It was agreed that everyone should be given the opportunity to contribute and that everyone's contribution should be valued. LGBTQ+ members emphasised a non‐heteronormative and trans‐inclusive approach and agreed that all co‐design members should be encouraged to put their pronouns in their on‐screen name. The care home staff group specifically mentioned that the meetings should be approached as a safe learning environment where everyone has the right intentions. These are interesting in the context of previous research, which suggests that older LGBTQ+ people experience care environments as hetero‐ and cis‐normative and fear discrimination, and care staff worry about using the ‘wrong’ language or approach and may act defensively [2, 13, 50]. This highlights the importance of meeting the groups separately to start the process in a way that is experienced as safe. These considerations were reintroduced at the start of the first collaborative session as an informal set of meeting terms.

In advance of the collaborative meetings, all co‐design members (including researchers) had written an informal biography about themselves, including information about why they joined the project, which helped to break down barriers between the groups. By outlining members' reasons for joining the project, the shared issue of a lack of LGBTQ+ inclusion in care homes and the shared goal of improving this were underlined.

#### 3.1.2 Development of New Resource

Below, we describe the processes that led to the final resource in the four collaborative meetings.

##### 3.1.2.1 Collaborative Meeting 1 (Discover and Define)

After reviewing existing resources, the group highlighted three main ways that the new resource should extend these. Firstly, co‐design members noted that most existing resources were too long to engage busy care home staff. A new resource that was not too heavy on text, prioritised inclusive actions as opposed to theory and background, and was usable in day‐to‐day practice was preferred. Secondly, the group commented on the specific formats needed to maximise the new resource's reach. Existing resources were predominantly available as online pages (printable) or videos. The group agreed early on that, ideally, the new resource would exist in different formats, so that it could be displayed/used in different ways but impart the same message. Thirdly, the group noted that whilst some previous resources include valuable tips on things care staff can do to make environments more inclusive, they did not involve all key aspects of a care journey (from looking for a care home to end‐of‐life care). The new resource aimed to function as a type of road map to the key experiences along the care journey.

##### 3.1.2.2 Collaborative Meeting 2 (Design)

The target audience was care home staff, but the group noted that the resource should be accessible enough to draw the attention of residents, family and other visitors too.

The group identified different formats to maximise engagement. For example, care home staff noted that screens in their reception areas could attract the attention of staff, residents and visitors, and a video or digital version of a leaflet/poster would work well. For staff supervision conversations, staff induction packs and more general conversation starters, something that exists in a physical form was thought to work well.

**Figure 2:**
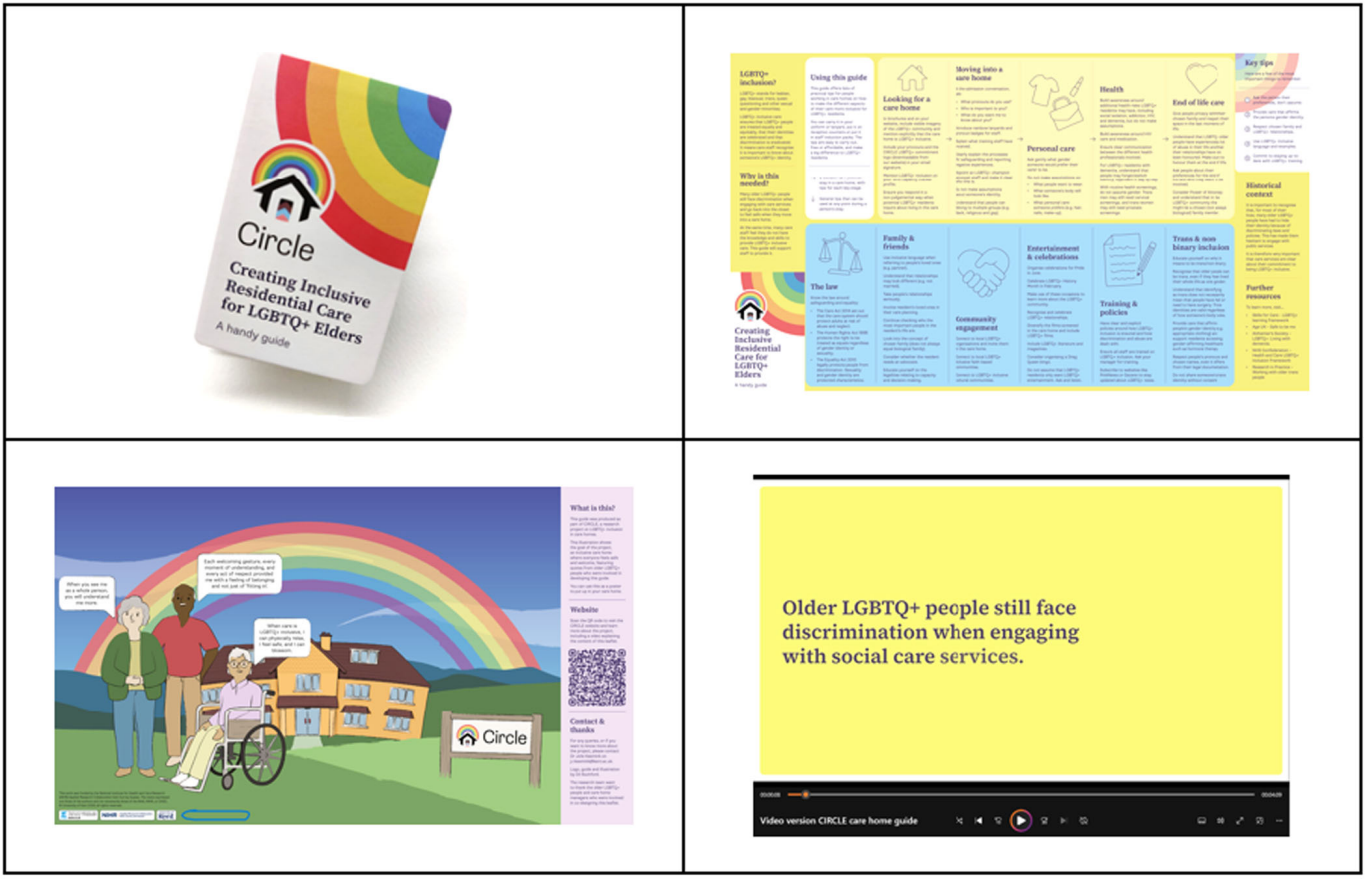
A preview of the different formats of the CIRCLE resource.

Early on, the ‘z‐card’ was brought up as a practical physical format. A z‐card is a compact, easy‐to‐use, foldable guide. Folded, it can be as small as a bank card; unfolded, it can provide a large amount of information, be read as a map or displayed as a poster (see Figure 2). The group agreed that its compactness was well‐suited to the care home environment and would easily fit into a uniform pocket. Unfolded, the z‐card could be displayed as a poster. We agreed not only to print hard copies of the z‐card (afforded by the CIRCLE budget) but also to publish the content in an easily printed online format. For accessibility reasons, it was agreed to also produce the z‐card content in video (audio‐visual) format.

The group also discussed the need for a recognisable logo that was used on all resource formats. It was felt the logo should have a clear and continuous style, so that it was clear what it represented (particularly important for the LGBTQ+ group with regard to their experienced invisibility). The group also discussed initial resource content ideas in this meeting, related to: (1) being careful with quantity of information to not overload people, (2) one side of the z‐card to include text‐based information and the other side an image, (3) a focus on positive actions for key experiences in the care journey and (4) referring to further resources.

##### 3.1.2.3 Collaborative Meeting 3 (Design)

Based on these initial ideas, the research team compiled a list of topics for the z‐card, including looking for a care home, moving into a care home, personal care, health, end‐of‐life care, the law, family and friends, community engagement, entertainment and celebrations, training and policies, and trans and non‐binary inclusion. During the meeting, the team spent time populating these topics with positive actions care home staff could take to make care experiences inclusive for LGBTQ+ residents. The challenge was to keep the action statements as short as possible, whilst maintaining informative value. It was also decided that, whilst the main focus should be on positive action, the z‐card should include a few short sentences on why this resource was needed and how it could be used.

The digital artist presented options for logos that the group provided feedback on. For the poster side of the z‐card, the group decided that the image should show a diversity of residents in front of an inclusive care home. The group suggested the residents have speech bubbles with quotes from LGBTQ+ co‐design members about how inclusive care makes them feel.

##### 3.1.2.4 Collaborative Meeting 4 (Deliver)

In the last meeting, the digital artist presented a draft version of the z‐card. The group's views were overwhelmingly positive, and they provided minor comments about small language amendments and the order of information. For the video version, it was decided that the information should be kept simple and that the main aim was to present key audio‐visual information. It was suggested that the video script would be read by co‐design members. Input on the final drafts of the z‐card and video were received via email due to time constraints.

#### 3.1.3 The Final Product

The final resource consists of: (1) a printed z‐card version of the care home guide, (2) a video presenting key z‐card content, (3) a printable booklet version of the care home guide and (4) a printable poster version of the care home guide. Figure 2 provides a preview of the final resource.

Whilst this resource was developed to extend previous resources, it is important to note that the co‐design team very much acknowledged the value of existing resources, such as, for example, the LGBTQ+ learning framework [26] and the hard work required to produce them. The new resource refers to existing resources as further reading for those who are interested and able to do more in‐depth reading on the topic.

The CIRCLE care home guide was officially launched during an online launch event in September 2024. It is freely available on https://research.kent.ac.uk/circle-guide/ after completing a registration form, allowing the team to track reach and impact.

### 3.2 Focus Group

This section summarises the findings of the content analysis of the focus group data on the perspectives of co‐design members on the co‐design process and outcome. The data have been synthesised following four identified content categories: (1) successes, (2) challenges, (3) collaboration and (4) impact.

#### 3.2.1 Successes

Co‐design members felt that their input was truly valued and reflected in the final product. They felt appreciated and included, and described the experience as ‘*being seen*’. This aligns with the principles that co‐design sets out to achieve [51] and was seen as a key success in this project.

For me, it was great being involved. I felt like it truly was a co‐design process. That it wasn't just lip service that was being paid to include those of us who are not researchers.

Older LGBTQ+ member

The achievement of producing an actual resource within the set time frame and budget was also viewed as a success. Decision‐making in a larger group can be challenging, and it can be time‐consuming to reach consensus. Co‐design members commented on how productive the group had been within a short time span. This demonstrates what can be achieved with limited time and budget, if the process is carried out efficiently.

How far we've come in a relatively short space of time, not knowing what our end result would be, and how quickly and enjoyably we've done that. It's been incredibly refreshing to have something tangible so quickly.

Care home staff member

It was noted that ‘*gentle shepherding’* by the research team had been paramount for productivity. So, whilst the research team aimed to be equal co‐design members in the process, the group appreciated a gentle steer to stay on track and within scope.

#### 3.2.2 Challenges

Whilst group members were overwhelmingly positive when speaking about their experiences of the co‐design process, some challenges had also been identified. Care home staff commented that it was not always easy to combine co‐design sessions with the demands of their care role. This meant they occasionally had to miss a session or leave meetings intermittently to deal with urgent matters. The research team adopted a flexible approach to accommodate this, which is recommended to enable care staff to be involved in similar projects.

Because when you're in a sort of living, breathing environment, it's really hard because residents walk in, staff members walk in, and then something happens down the corridor, or there's some kind of crisis. So that was the only obstacle really, but you're always gonna get that in a care home environment, because it's such a busy environment.

Care home staff member

Some of the older LGBTQ+ members mentioned the challenges they sometimes experienced with the meeting technology. However, they also noted that they were able to join the co‐design process because of it being remote. A patient and understanding attitude from the research team was appreciated.

#### 3.2.3 Collaboration

It can be challenging bringing different perspectives together, especially in this case where there was potential historical tension between groups, and also, as the group highlighted, uniting different perspectives within the LGBTQ+ community. Members commented on how well this had gone in the current project. They found it valuable to unite the different perspectives (LGBTQ+ lived experience and professional care practice) and believed it helped balance the subject expertise and usability for practice. The group appreciated the diversity of ideas, and having a shared goal worked effectively to achieve the outcome.

l loved that aspect. It showed us that we were looking at the same picture but seeing different views, not right or wrong, just different. It made the project richer in terms of information gathered and tested the relevance of product during the process.

Care home staff member

Some older LGBTQ+ members mentioned the importance of facilitating separate initial meetings, but then moving towards collaborative meetings. This had helped create an initial sense of safety and further ensured that the end product was a truly collaborative piece of work representing all stakeholders' views.

I was pleased for your sensitivity that you had brought the two groups together separately to get the chance to know the other folk in my group, quote unquote, before sort of the wider group, but I thought it worked brilliantly, combining both groups to come to the subject from different perspectives with different experiences. If the groups were kept separated then you might have ended up with an elephant and a giraffe. Whereas at least we have a, I don't know, a spotty elephant.

Older LGBTQ+ member

The care home staff also highlighted how essential it had been to work together with a group of older LGBTQ+ people to learn more from people with lived experience.

#### 3.2.4 Impact

The impact of the process can be expressed in two ways. Firstly, the impact is on the people involved and how the process has shaped them. Some older LGBTQ+ members mentioned the positive impact of the opportunity to be involved in research and to connect with others they would not normally meet.

I think the main thing that I've got out of it is, I've never been involved in a virtual research group before. And it's been a very positive experience. And I wouldn't have got to meet any of you had it not been for being part of it. So I'm really, really glad I tripped over the invitation.

Older LGBTQ+ member

Care home staff mentioned how their involvement had led to changes in their care practice and their understanding of LGBTQ+ inclusion in care. Examples of changes in care practice included the organisation of Pride celebrations, changing assessment forms to include more inclusive language and feeling empowered to ask more open questions. One person mentioned they had learned about true allyship, illustrated by the quote below.

Doing nothing is not an option, you have to be active in your support of the LGBT community.

Care home staff member

The research team also discussed the project's personal impact, noting feelings of encouragement and hope engendered by the co‐design process. Their initial apprehensions about balancing shifting power dynamics between groups proved unfounded, with reassuring evidence that group members found the co‐design a positive process.

Secondly, the anticipated impact of the co‐designed resource is also discussed. The group was hopeful about the resource's potential impact. Care staff emphasised they were excited to introduce the resource to their staff and could envision how it could benefit their practice. All members hoped that the resource would be used widely.

I would love to see a z‐card in every pocket of every member of staff's uniform, I would love to see it become really dog tagged and really torn apart, because that means that people are using it.

Older LGBTQ+ member

There were also some concerns about the potential sustainability of the impact. The group acknowledged that new resources and ideas are launched regularly, garnering only short‐term attention and impact. However, the z‐card's content is not time‐limited, potentially prolonging its longevity. Care home staff also emphasised the importance of care sector leaders recognising that the focus on LGBTQ+ inclusion must be continued and that they have a key role to play in achieving that.

I think as managers and leaders of the care home sector, it's our responsibility to keep that message alive.

Care home staff member

Future impact data will be needed to demonstrate the actual impact of the resource in practice.

## 4 Discussion

This paper is the first to report on the development of a novel, practice‐focused resource on LGBTQ+ inclusion for care homes, co‐designed by older LGBTQ+ people, care home staff and researchers. Many older LGBTQ+ people still face discrimination in care home settings [9, 52], and many care home staff do not have the knowledge required to provide LGBTQ+ inclusive care [4]. Despite the availability of valuable resources on LGBTQ+ inclusion, widespread use in social care practice is lacking [22]. We identified that existing resources present some shortcomings, such as the effort and time required to engage with them, a lack of techniques that support the intended audience in changing their behaviour, formats not aligning with audience needs, and insufficient co‐design with key stakeholders. The resource presented here extends existing resources by being fully co‐designed by older LGBTQ+ people, care home staff and researchers. It also prioritises positive actions that care staff can take throughout the care journey in time‐efficient formats designed to work well in care home settings.

This study demonstrated that older LGBTQ+ people, care home staff and researchers can successfully collaborate in a facilitated EBCD process to produce a resource intended for care service improvement. Our findings highlight the power of lived experience, both from an older LGBTQ+ person's and care staff's perspective. Both perspectives brought unique elements to the co‐design sessions and are represented in the final resource. The EBCD method seemed to work well in this context as it is well‐suited to bringing groups with different perspectives together in a productive, solution‐focused way [44]. There are very few examples of collaboration between care home staff and older LGBTQ+ people in the literature [37], despite several researchers underlining the need for this [32, 33, 34, 35]. The current study contributes to this limited knowledge base and highlights the benefits of collaboration. Whilst the researchers in Willis et al.'s [37] study focused more on the co‐production of research, rather than co‐designing a practice resource, we can draw some parallels between our findings. Both studies underline the importance of the inclusion of lived experience, as well as using different types of expertise to create change. Further research and service improvement projects can build on this knowledge.

In addition to presenting a novel resource for care homes co‐designed by older LGBTQ+ people, care home staff and researchers, this study also contributes to the literature by reporting on participants' experience of a co‐design process. Although the method of co‐design is widely used, there is limited literature that reports or evaluates the process and impact [36, 53]. The current paper reports the co‐design process in a detailed manner, and moreover, gathered data on co‐design members' experiences. Our findings demonstrate the positive impact of the co‐design process, with participants reporting feelings of being included in a meaningful way and making new social connections. Interestingly, care home staff also reported that being involved in this co‐design process had directly impacted their care practice to reflect more LGBTQ+ inclusive care values. This suggests that collaboration between these two groups can function as an educational experience on LGBTQ+ inclusion in itself, which confirms the power of lived experience in inclusion training and aligns with previous research [22, 54]. Furthermore, this has wider implications for the design of care home services in general, as it illustrates the impact of meaningful involvement of (prospective) care recipients in service design, aligning with the UK government's recommendation for co‐production of care services [55]. Co‐design of services embodies person‐ and relationship‐focused care and can offer unique insights essential for care delivery [56].

We acknowledge that the current study presents a very positive picture of this co‐design process. This is partly a genuine reflection of the experience of the co‐design members, but it is also important to recognise what factors may have contributed to the positive experience, as well as considering potential limitations to this study. Reflecting on the positionality of the researchers, it is worth noting that some of the research team identify as LGBTQ+ themselves. Previous research suggests that researchers' direct experience of participant identity may support relinquishing traditional hierarchies [57], which may be particularly relevant in co‐design studies. Further, the team had existing connections with some of the co‐design members through community work, which may have enhanced their comfort. Additionally, some of the co‐design members (both lived experience and care home staff members) had been involved in research or LGBTQ+ inclusion work before, which may have benefited the efficiency of the process. We do not view these factors as limitations; rather, they could be seen as strengths. The suggestions described here can be useful considerations for future similar projects.

One limitation of this study is a relative lack of diversity within the co‐design team. Although there was diversity in terms of sexuality, disability and age, the team was predominantly white British and cisgender. We tried to mitigate this by acquiring additional input on the resource from a trans expert‐by‐experience. In the resource, we describe the importance of awareness of intersecting minority identities and their experiences; however, we cannot guarantee that the lived experiences and views of all minority groups and intersecting identities are truly represented. This is an important consideration for future work, and time and resources should be allocated to maximise diversity. Secondly, whilst the use of Zoom in this study was experienced as a benefit, it is important to acknowledge that this may have precluded participants with poor digital skills and confidence and those without (reliable) internet connection [58]. We supported our participants who indicated low digital confidence by offering pre‐meetings and explanations of the technology. However, we did not offer face‐to‐face meeting options, which possibly excluded potential participants. With regard to methodological limitations, we do not present actual impact data on the resource in this paper. The resource was launched in September 2024, and the initial 20,000 physical copies have been distributed to care homes in several Local Authorities supporting the project and will be available at regional and national care conferences. The downloadable versions of the resource are promoted by several national care workforce organisations. Although the resource was designed to suit care home practice and the co‐design process itself was a success, the success of the resource will largely depend on how well it is adopted by care staff. Hafford‐Letchfield et al. [11] emphasise the importance of giving attention to *how* resources are used in addition to the information they provide. We are working on a follow‐up study on the reach and impact of the resource to demonstrate resource usability and potential practice change. This will be reported in a future paper.

## 5 Conclusion

Through an inclusive EBCD process, we developed a new resource for care homes to support LGBTQ+ inclusive practice. The resource is unique in that it was fully co‐designed by older LGBTQ+ people, care home staff and researchers. It prioritises positive actions and is available in formats designed to work well in a care home environment. The co‐design group reported positive experiences about collaborating as older LGBTQ+ people and care home staff, on which future projects can build. The final version of the resource has been launched, and future research will assess its impact in practice.

## Author Contributions

**Jolie R. Keemink:** conceptualisation, investigation, funding acquisition, writing – original draft, methodology, supervision, formal analysis, project administration, visualisation, data curation, resources. **John Hammond:** conceptualisation, writing – review and editing, investigation, resources. **Grace Collins:** writing – review and editing, investigation, methodology, resources. **Joseph Price:** formal analysis, data curation, investigation, writing – review and editing. **Martin Wells:** investigation, writing – review and editing. **Sallie Johnson:** investigation, writing – review and editing. **Susan A. Rugg:** investigation, writing – review and editing. **Martin Parish:** investigation, writing – review and editing. **Andrew King:** conceptualisation, funding acquisition, writing – review and editing, supervision, validation. **Kathryn Almack:** supervision, writing – review and editing, conceptualisation, funding acquisition, validation.

## Ethics Statement

This study was approved by the Ethics Committee of the School for Social Policy, Sociology, and Social Research at the University of Kent in November 2022 (Ref: SCREA ID 0722). All participants provided written consent to participate in the co‐design process, have the co‐design session recorded and participate in the focus group. At the start of each co‐design session, all members were asked if they were still happy to be involved.

## Conflicts of Interest

The authors declare no conflicts of interest.

## Data Availability

The data that support the findings of this study are available from the corresponding author upon reasonable request.
